# Disease-generic factors of work participation of workers with a chronic disease: a systematic review

**DOI:** 10.1007/s00420-015-1025-2

**Published:** 2015-02-25

**Authors:** Marloes Vooijs, Monique C. J. Leensen, Jan L. Hoving, Joost G. Daams, Haije Wind, Monique H. W. Frings-Dresen

**Affiliations:** Amsterdam, The Netherlands

**Keywords:** Chronic illness, Chronic condition, Health status, Occupational health, Employment, Stay at work

## Abstract

**Purpose:**

The purpose of this review was to search systematically for disease-generic factors associated with either work retention (WR) or return to work (RTW) in people of working age with a chronic disease.

**Methods:**

An extensive search was performed in PubMed, EMBASE, PsycINFO and CINAHL for English-, Dutch- and German-language studies searching on synonyms of the terms chronic disease, WR and RTW. Studies were selected if they described factors related to WR or RTW and included participants with a chronic disease of working age (15–67 years old).

**Results:**

From 2597 hits in the electronic databases, we identified six studies reporting 23 factors associated with work participation. Categorized according to the International Classification of Functioning, Disability and Health, health-related factors (comorbidity, duration of symptoms and less dysfunction), environmental factors (work environment and duration of absence) and personal factors (age, gender, education and own prediction of RTW) were identified.

**Conclusions:**

Various disease-generic factors are associated with work participation, of which most of the reported factors are independent of diagnosis. Evidence of the retrieved factors is restricted due to the limited availability of studies focusing on disease-generic factors and overall low quality of the retrieved studies.

## Introduction

Chronic diseases, defined by the World Health Organization as “diseases with long duration and generally slow progression” (WHO 2014), are the leading cause of morbidity worldwide (WHO 2008). In 2011, approximately 29 % of the male population and 34 % of the female population aged 16 years or over in the European Union reported having a chronic illness. In the working population, the prevalence of having one or more chronic diseases ranges from 10 % (16–24 years) to 55 % (55–64 years; Eurostat 2014). Due to enhanced treatment, which improved the survival rates of patients with various diagnoses (Baan and Schoemaker 2009), and an increase in incidence of diseases due to unhealthy lifestyles (WHO 2002), increasing numbers of people in the working population are affected by one or more chronic diseases.

Having work is beneficial for health status, since it improves functional outcomes, social integration and satisfaction with life status and financial status (Kirsh et al. 2009). Previous studies showed that having a chronic disease affects work participation negatively; people with a chronic disease are less often employed (Australian Institute of Health and Welfare 2009; Maurits et al. 2013) and, when they are employed, work on average fewer hours (Koppes et al. 2012) than the general population does. In addition, employees with a chronic disease report having difficulties meeting work demands (Koppes et al. 2012; Koolhaas et al. 2013). If, however, factors that hinder or promote work retention (WR) and return to work (RTW) could be identified, these factors could be considered in interventions whose aim is to improve work participation.

WR focuses on preventing work loss in workers with a chronic disease. This is important because employees experience RTW as being difficult once absent from work (Noordik et al. 2011; Kuijer et al. 2006). However, sometimes sickness absence is inevitable which is, if possible, followed by re-entry in the same job or a different one after a period of sickness absence. Encouragement and early intervention in targeted subgroups of workers are important factors, since the longer the sickness absence lasts, the less likely people are to RTW (Peters et al. 2007).

Previous research has shown that some people manage to stay at work or return to work, where others with the same disease and prognosis do not (Van Muijen et al. 2013; De Vries et al. 2012; Achterberg et al. 2009). This indicates that besides disease-related factors, other factors could influence work participation of patients with various diagnoses, i.e., disease-generic factors. This is reflected in the ICF that describes mutual interactions between six different dimensions, showing that participation is not only affected by disease-related factors but also affected by personal and environmental factors, which are independent of diagnoses (WHO 2001). A previous review did address these disease-generic factors in relation to work disability, in which it was found that perceived complaints, limitation in physical activities, heavy manual work and female gender were associated with work disability (Detaille et al. 2009).

In this systematic review, we want to broaden the applicability of disease-generic factors by placing no limit on the chronic diseases to be included. Instead, we searched for studies that examined study populations with a variety of chronic diseases. Moreover, to our knowledge, no systematic review has been previously conducted in order to search for disease-generic factors associated with WR or RTW specifically. The purpose of this systematic review is therefore to answer the following question: Which disease-generic factors are associated with WR or RTW of people of working age with a chronic disease?

## Materials and methods

During the development of this review, we strived to address all items reported in the PRISMA (Preferred Reporting Items for Systematic Reviews and Meta-Analyses) statement (Moher et al. 2009).

### Search strategy

The literature search aimed to identify all published papers that studied factors associated with WR or RTW in people of working age with a chronic disease. The first author (MV) and an experienced clinical librarian (JD) performed an extensive search in March 2014 in PubMed, EMBASE, PsycINFO and CINAHL, using MeSH terms, subheadings and free text words. Since our aim was to retrieve studies, which included a study sample with various diagnoses, we searched on synonyms of the term “chronic disease,” in combination with terms related to the outcome variables. A full description of the literature search is presented in Appendix 1. The strategy was formulated in PubMed (MEDLINE) and was adapted for the use in EMBASE (OvidSP), PsycINFO (OvidSP) and CINAHL (EBSCOhost). The search was limited to articles with a publication date ranging from January 2004 to March 2014. The references of all included studies were screened for additional relevant publications, which were checked according to the original search terms in order to retrieve studies with a study sample of various diagnoses.

### Selection of studies

Citations and abstracts of all studies were retrieved, and duplicates were removed. Selection of the studies was performed in two rounds; the first round consisted of the title and abstract screening in which the first author (MV) screened all the retrieved records. Four authors (ML, JH, HW and MF) each screened a quarter of the records independently regarding whether the records reported a chronic disease, used an adequate study design and used WR or RTW as an outcome. If the title and abstract failed to meet one or more selection criteria, the publication was excluded. When there was no sufficient information in the title and abstract to judge eligibility, the full-text article was retrieved. In the second round, full-text articles were ordered and studies were selected based on all defined criteria by the first author (MV) and second author (ML). We included reviews, cohort studies (both prospective and retrospective), cross-sectional studies and case–control studies, which searched for factors associated with the outcomes WR or RTW. We defined WR as preventing work loss or staying employed. RTW was defined as re-entering employment in the same job or a different one after a period of sickness absence. We included studies in which the participants were of working age (15–67 years) and had a chronic disease for more than 3 months, following the definition of chronic disease according to the National Centre for Health Statistics (2010). Only papers written in English, Dutch or German to which we had access to both abstract and full-text article were considered for inclusion in this review. The original studies of the reviews which were included in full-text selection were retrieved and screened on title and abstract and, if the selection criteria were met, on full text. Disagreements during the process of selecting were resolved by obtaining consensus during a weekly meeting with the reviewers. For practical considerations, papers were not blinded for authors, institutions, journal, results or conclusions.

### Quality assessment

Two reviewers (MV and JH) independently scored the quality of the included studies using an adapted version of the Methodological Evaluation of Observational Research checklist (Shamliyan et al. 2011), derived from Robroek et al. (2013) and Ijaz et al. (2013). Criteria addressed were study design, loss of follow-up or non-response, standardized or valid measurement of both outcomes and factors, measurement of confounding factors and methods to reduce bias. When the criterion was sufficiently met, it was scored as 1. When the criterion was not sufficiently met or not reported, it was scored as 0. It was decided that the study had to meet four of the six criteria in order to obtain the label “of sufficient quality.” Disagreements between the two reviewers were resolved through consensus. If agreement was not reached, the fifth author (HW) made the final decision.

### Data extraction

The first reviewer (MV) performed the data extraction using a standardized form that included items on demographic characteristics of the study population (age, gender and chronic disease), study design, sample size, outcome measures concerning WR and RTW, factors associated with outcome and estimated effect size. Data extraction was checked by four reviewers (ML, JH, HW and MF). When performing the data extraction, we reported the associations observed in the multivariate model. When a prediction model was used, the univariate associations were reported in order to retrieve the independent associations. When multiple models were estimated for different outcomes, we used the model that matched our outcome as closely as possible. Data were extracted for all factors, including the factors that were specifically aimed at one specific disease (e.g., “primary type of dialyses”). However, it was decided not to include this data in the further description of the results. The data extraction can be found in Appendix 2.

## Results

### Selection of studies

**Fig. 1:**
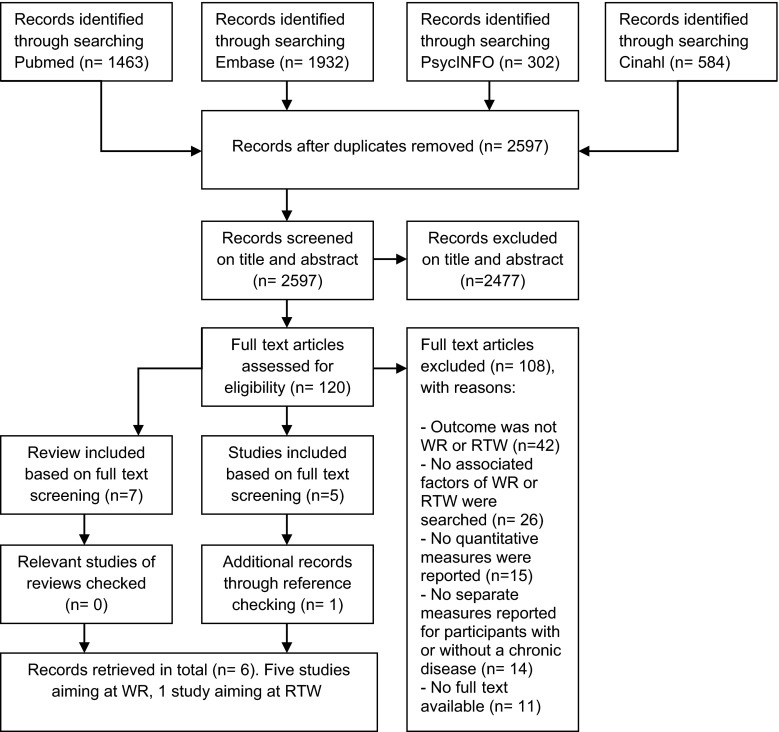
Flowchart selection of studies

The search yielded 4,281 unique records: 1,463 from PubMed, 1,932 from EMBASE, 302 from PsycINFO and 584 from CINAHL. After duplicates had been removed, 2,597 articles were identified. Based on title and abstract, 2,477 articles were excluded, mostly because their outcomes did not match WR or RTW. From the 120 remaining articles, five studies and seven reviews were selected. Checking the original studies of the included seven reviews did not yield any additional studies. Reference checking of the five included studies revealed one new article. This resulted in a total of six studies that met the inclusion criteria and were included in this review, five studies with WR as their focus and one study whose topic was RTW. The results of the literature search are presented in Fig. 1. The summary of the methodological ranking for each study is presented in Table 1. As can been seen from Table 1, of the six studies, two studies were rated as sufficiently meeting the quality criteria.

**Table 1:** Quality assessment of the six included studies

Author (year)	Design			Outcome	Factors	Confounding	Analysis	Total
	1^a^	2a^b^	2b^c^	3^d^	4^e^	5^f^	6^g^	Sufficient quality or insufficient quality
Botticello et al. (2012)	0	NA	0	1	1	1	1	Sufficient
Calsbeek et al. (2006)	0	NA	0	1	1	0	0	Insufficient
Heijbel et al. (2006)	1	1	NA	1	1	0	0	Sufficient
Messmer Uccelli et al. (2009)	0	NA	0	NR	0	0	0	Insufficient
Muehrer et al. (2011)	1	0	NA	0	0	0	0	Insufficient
Baanders et al. (2002)	0	NA	0	NR	1	1	1	Insufficient

*NA* not applicable, *NR* not reported

^a^Cohort design: 1, other than cohort design, unclear or not reported: 0

^b^Number of dropouts/loss to follow-up ≤20 %: 1, number >20 %, unclear, not reported or other study design: 0

^c^≤20 % of non-response differed among cases and controls: 1, >20 % of non-response differed among cases and controls or >20 % of non-response reported for cases only, unclear, not reported or other study design: 0

^d^Outcome measures are measured in a standardized or valid way: 1, outcome measures are measures in a non-standardized or non-valid way, unclear, not reported: 0

^e^Factors are measured in a standardized or valid way: 1, factors are measures in a non-standardized or non-valid way, unclear or not reported: 0

^f^Major confounding factors were assessed in full and measured in a validated way: 1, major confounding factors were not assessed, unclear or not reported: 0

^g^Authors reported using methods to reduce bias: 1, authors did not use methods to reduce bias, unclear, not reported: 0

### Data analyses and outcomes

Regardless of the analyzing methods used, all studies reported one or more factors statistically significantly associated with the outcomes WR and RTW. As data analyses varied considerably, direct comparisons between studies presenting absolute point estimates and studies presenting regression parameters are less informative. We considered the pooling of the results as not being useful, due to the heterogeneity in study quality and studied factors between the studies. For this reason, we evaluated the results of the study in a qualitative way and described the factors according to the ICF model.

#### Work retention

Five studies were retrieved regarding WR, of which one study was of sufficient quality. Factors associated with WR are listed in Table 2.^1^ Regarding the ICF dimension of personal factors, two studies found that female gender (*p* < 0.01^a^, neg.; OR 0.78, 95 % CI 0.74–0.81) and older age were negatively associated with WR. Age reduced the chance of WR when being over 55 years old (55–59 years old, OR 0.87, 95 % CI 0.82–0.93 and 60–64 years old, OR 0.89, 95 % CI 0.82–0.97) and being 20–24 years of age (OR 0.85, 95 % CI 0.75–0.97). On the other hand, being 25–44 years old was positively associated with WR (*p* < 0.01^a^). Also, a lower educational level, race, substance use, use of medication and nocturnal toilet use were found to be negatively associated with WR. Having a higher socioeconomic status (SES) index was positively associated with WR. Other factors associated with WR, using the ICF model, were comorbidity and experiencing motor control problems (body function/structure dimension). Also, inability to ambulate (activity dimension), living in an urban area, workplace environment and financial considerations (environmental dimension) were reported to be associated with WR.

**Table 2:** Study and patient characteristics of the six included studies

First author, year, (reference), country of origin	Heijbel et al. (2006), Sweden	Baanders et al. (2002), the Netherlands	Botticello et al. (2012), USA	Calsbeek et al. (2006), The Netherlands	Messmer Uccelli et al. (2009), 18 European countries	Muehrer et al. (2011), USA
Study design	PC	CS	CS	CS	CS	RC
Sample size	508	1,266	1,013	246	1,141	102,104
Gender (F %)	90.9	56.5	19.1	58.2^b^	67	NR
Range of age (mean, SD)	24–64 (50^a^, NR)	15–64 (NR, NR)	17–64 (41.2, NR)	15–24 (20.1, NR)	21–67 (41.8, 9.2)	15–64 (NR, NR)
Chronic disease	Musculoskeletal pain (33.7 %^b^), mental distress (15.6 %^b^), respiratory disorders (1.9 %^b^), cardiovascular disorders (1.5 %^b^), other (i.e., neurological disorders, factures, diabetes, 12.0 %^b^), combination of disorders (35.2 %^b^)	Cardiovascular disease (7.7 %), chronic nonspecific lung diseases (18.6 %), locomotor disease (15.0 %), cancer (5.1 %), diabetes mellitus (10.4 %), neurological disease (9.1 %), digestive disorder (3.5 %), other (30.6 %)	Spinal cord injury; paraplegia (53.2 %), tetraplegia (46.8 %)	Inflammatory bowel disease (49.2 %), chronic liver diseases (11.7 %), congenital digestive disorder (17.6 %), food allergy (9.4 %), celiac disease (12.1 %)	Multiple sclerosis	Chronic kidney disease, end-stage renal disease (ESRD)
Employment (%) at baseline	100	45.1	63.4	NR	61	NR
**Outcome**						
Definition	RTW, defined as work status on the 18th month after baseline. Persons who had returned (part-time) and were working for <15 days during the 18th month were counted as returners	WR, defined as labor market participation is defined as having a paid job for at least 12 h per week	WR, defined as employment status, assessed at least one follow-up. The variable was dichotomized as ‘paid employment’ ‘yes’ (category: ‘working’) and ‘no’ (remaining categories)	WR, defined as labor participation, assessed by the number of hours employed per week which were dichotomized in ‘having a paid job’ (< 12 h/w) vs. ‘not having a paid job’)	WR, defined as employment status; differentiating between employed or not employed.	WR, defined as maintaining employment. Person’s inability to maintain employed was identified when, at initiation of treatment, persons changed employment from full- to part-time or to any other status
Measurement	Human Resources departments in 5 municipalities and 4 county councils	Postal questionnaire	Telephone interview	Postal questionnaire	Questionnaire	US Renal Data System (USRDS)
**Factor**						
Factor	1. Age (UV) •≤44 years •45–54 years 2. Own pos. prediction RTW 3. Complaints >1 group symptoms 4. Duration complaints ≤5 years 5. Duration sick leave <1 years 6. Less pain: •1st quartile (r: 4th quartile), •2nd quartile (r: 4th quartile) •3rd quartile (r: 4th quartile) 7. Less impairment: •1st quartile (r: 4th quartile) •2nd quartile (r: 4th quartile) 8. Perception welcome at work	1. Female gender 2. Age (25–44 years) 3. Lower educational level: •Primary •Lower 2nd + vocational 4. Motor control problems	1. >Socioeconomic status (SES) index 2. Urban living	1. Use of medication (UV) 2. Nocturnal toilet use	1. Workplace environment 2. Financial considerations	1. Age: (MV) •20–24 years •55–59 •60–64 2. Female gender: 3. Race: •Black •Asian •Hispanic •Other 4. Substance use: •Alcohol use •Use of drugs •Tobacco use 5. Comorbidity: •Coronary vascular dis •Cancer •Congestive heart failure •Ischemic heart disease •COPD •Cardiac arrest •Hypertension •Diabetes (insulin) 6. Inability to ambulate
Effect	1. •2.48 [1.43, 4.31] •2.18 [1.30, 3.67] 2. 15.99 [6.86, 37.25] 3. 2.01 [1.29, 3.13] 4. 1.75 [1.12, 2.72] 5. 2.67 [1.76, 4.05] 6. •3.73 [1.84, 7.55] •5.51 [2.74, 11.07] •2.23 [1.06, 4.70] 7. •2.70 [1.41, 5.16] •2.05 [1.06, 3.97] 8. 1.92 [1.23, 2.99]	1. *p* < 0.01, neg. 2. *p* < 0.01, pos. 3. •*p* < 0.01, neg. •*p* < 0.01, neg. 4. <0.01, neg.	1. 1.09 [1.04, 1.14] 2. 0.46 [0.23, 0.93]	1. 0.78 [0.62, 0.98] 2. 0.70 [0.53, 0.91]	1. 1.04 [1.01, 1.06] 2. 1.15 [1.07, 1.23]	1. •0.85 [0.75, 0.97] •0.87 [0.82, 0.93] •0.89 [0.82, 0.97] 2. 0.78 [0.74, 0.81] 3. •0.75 [0.72, 0.78] •0.82 [0.74, 0.90] •0.68 [0.65, 0.71] •0.85 [0.73, 0.98] 4. •0.56 [0.47, 0.67] •0.73 [0.60, 0.89] •0.85 [0.79, 0.93] 5. •0.70 [0.62, 0.79] •0.73 [0.65, 0.81] •0.80 [0.76, 0.85] •0.92 [0.85, 0.99] •0.86 [0.75, 0.98] •0.55 [0.39, 0.76] •1.06 [1.00, 1.11] •0.94 [0.89, 1.00] 6. 0.45 [0.35, 0.58]
Measurement	Postal questionnaire (*n* = 520), telephone interview (*n* = 5)	Postal questionnaire	National Historical Geographical Information System, 2000 US Census Summary File	Postal questionnaire.	Questionnaire.	b) US Renal Data System (USRDS)

*PC* prospective cohort, *RC* retrospective cohort, *CS* cross-sectional study, *UV* univariate measurement, *MV* multivariate measurement, *NR* not reported, *r* reference category

^a^Median of population at baseline

^b^Statistics of population at baseline

#### Return to work

In the one study using RTW as an outcome, having a younger age (OR 2.48, 95 % CI 1.43–4.31) and the sick-listed persons’ own prediction of their RTW (≤44 years old, OR 15.99, 95 % CI 6.86–37.25) were reported to be positively associated with RTW. Other factors associated with RTW, in terms of ICF dimensions, are as follows: complaints from not more than one group of symptoms, duration of complaints <5 years, less pain and less impairment (body function/structure dimension), shorter duration of sick leave (participation dimension) and, regarding the environmental dimension, the perception of feeling welcome back at work (see Table 2).

## Discussion

The aim of this systematic review was to retrieve disease-generic factors associated with WR or RTW of workers with a chronic disease. We identified several factors associated with WR or RTW across all ICF dimensions. Of these results, factors reported in multiple studies were age and gender. The patient’s own prediction of RTW was found to have a large effect on RTW in one study.

Both older age and female gender, relating to the personal dimension of the ICF, were reported to be negatively associated with work participation, which is consistent with the findings of other systematic reviews (Van Muijen et al. 2013; De Vries et al. 2012), focusing on specific diseases. The systematic review of Detaille et al. (2009), focusing on prognostic factors of work disability common in the five most prevalent chronic diseases (rheumatoid arthritis, asthma, chronic obstructive pulmonary disease, diabetes mellitus and ischemic heart disease), found a negative association of both older age and female gender with work disability. Since our results are in line with these previous studies, despite the different outcome parameters and study populations, this would indicate that the associations of older age and female gender with work participation are independent of diagnosis. This supports our hypothesis that factors other than disease-related factors play a significant role in WR or RTW of the chronically ill.

Age was reported by several studies in this review (Heijbel et al. 2006; Baanders et al. 2002; Muehrer et al. 2011), with the most consistent finding of older age being negatively associated with work participation. Fraser et al. (2009) reported that older workers can experience age discrimination and consider this a barrier for work participation. The negative association of female gender with work participation (Baanders et al. 2002; Muehrer et al. 2011) was explained by Côté and Coutu (2010) by how men and women perceive themselves in relation to their social environment, i.e., social identity. Work-associated self-identity may foster social stereotyping of gender roles, especially that of the man as breadwinner (Ghaill and Haywood 2007), which may influence the higher chance of RTW for men. Given the aging working population, the increasing work participation by women and the trend that people will have to work longer before their retirement in Western countries (Crepaldi et al. 2008), a substantial part of the workers will be at risk for reduced work participation. As these personal factors, age and gender, are not modifiable, more intensive guidance at an early stage targeted at these higher-risk groups could be implemented to enhance future work participation.

With regard to the association of one’s own prediction of RTW and work participation, Heijbel et al. (2006) reported that the predictive value of a person’s own negative prediction regarding RTW was 96 %. This means that only 4 out of 100 people with a negative prediction does in fact RTW after sickness absence. This result is in line with previous research, indicating that the prediction of RTW is an important indicator of RTW (Cole et al. 2002). In addition, the study of Wind et al. (Wind et al. 2013) showed that patients are capable of predicting their own RTW in the context of disability claims. Dunstan et al. (2013), which operationalized the prediction of RTW by the term “Behavioral Intention” (BI), states that BI can be influenced by a change in how one thinks about work, how the social environment thinks about RTW and how one perceives the behavior, RTW, to be under his or her control. With regard to the social environment, Dunstan et al. (2013) reported that the doctor’s opinion carried the greatest weight and therefore influences the patient’s expectation of RTW, meaning that health professionals should bear in mind that their opinion influences the RTW of their patients. In addition, expectation of RTW is subject to change by altering the patient’s attitude about work and the perception of feeling in control of their own behavior of RTW (Dunstan et al. 2013), these being the two other components of BI. By identifying workers with a negative prediction of their RTW at an early stage, and aiming specific interventions at these groups, work participation could be enhanced.

This systematic review revealed that studies including study populations with various diagnoses are limited. Therefore, in addition to the low overall quality of the retrieved studies, evidence of the factors associated with work participation is restricted. The factors retrieved in this review, i.e., age, gender and prediction of RTW, are among the most commonly reported factors associated with work participation. This review shows that these factors are applicable to populations with various diagnoses. These disease-generic factors provide insight for health professionals who are at risk for reduced work participation. One should keep in mind that participation in work could also be affected by factors dependent on the type of diagnosis. For example, treatment-related factors, such as the adverse effects of intensive chemotherapy (Taskila and Lindbohm 2007), can influence work participation in workers with cancer. Both disease-generic and disease-specific factors can be targeted to optimize work participation efforts.

Further research should aim to increase the evidence regarding disease-generic factors associated with work participation in chronically ill workers, additional to those identified in our review. These factors could help professionals involved in work participation programmes to identify workers who are at high risk of not participating in work and to target interventions early in the process in order to enhance work participation.

## Conclusion

The objective of this review was to search systematically for disease-generic factors associated with either WR or RTW in people of working age with a chronic disease.

Various disease-generic factors are associated with work participation, of which most of the reported factors are independent of diagnosis. Evidence for the retrieved factors is restricted, due to the limited availability of studies focusing on disease-generic factors and the overall low quality of the studies.

## Appendix 1: Search strategy

### PubMed, Date of search February 27, 2014

(“chronic disease”[Mesh] OR chronic disease*[tw] OR chronic disorder*[tw] OR chronic health[tw] OR chronic condition*[tw]) AND (“return to work”[Mesh] OR (return to[tw] AND work[tw]) OR back to work[tw] OR unemployment[Mesh] OR unemployment[tw] OR “Employment”[Mesh:NoExp] OR employment[tw] OR employability[tw] OR work resumption[tw] OR working age[tw] OR “job satisfaction”[Mesh] OR “sick leave”[Mesh] OR absenteeism[Mesh] OR sick leave[tw] OR absenteeism[tw] OR work retention[tw] OR job retention[tw] OR job status[tw] OR work status[tw] OR employment status[tw] OR paid work[tw] OR vocational status[tw] OR occupational status[tw] OR work functioning[tw] OR job functioning[tw] OR work capacity[tw] OR employment capacity[tw] OR work participation[tw] OR employment participation[tw] OR stay at work[tw] OR presenteeism[tw] OR work outcomes[tw] OR work ability[tw]).

Note: no additional limits have been applied.

### EMBASE Classic + EMBASE 1947: Present (OvidSP), Date of search March 4, 2014

1. chronic disease/
2. (chronic illness or chronic disease* or chronic disorder* or chronic condition or chronic health). ab, kw, ti.
3. return to work/
4. (return to work or (return to adj3 work) or back to work). ab, kw, ti
5. unemployment/
6. unemployment. ab, kw, ti
7. employment/
8. (employment or employability). ab, kw, ti
9. employment status/
10. (employment status or job status or work status or vocational status or occupational status or paid work). ab, kw, ti
11. work resumption/
12. (work resumption or working age or work retention or job retention or work functioning or job functioning or work participation or employment participation or stay at work or presenteeism or work outcomes). ab, kw, ti
13. work capacity/
14. (work capacity or employment capacity or work ability). ab, kw, ti
15. job satisfaction/
16. job satisfaction. ab, kw, ti
17. absenteeism/
18. (absenteeism or sick leave). ab, kw, ti
19. or/3–18 [RTW or job retention]
20. 1 or 2 [chronic diseases]
21. 19 and 20

Note: no additional limits have been applied.

### PsycINFO 1806 to Present (OvidSP), Date of search March 5, 2014

1. ”chronicity (Disorders)”/or “chronic illness”/
2. (chronic disease or chronic disorder? or chronic health or chronic condition or chronic illness). ab, id, ti
3. reemployment/
4. (return to work or (return to adj3 work) or back to work). ab, id, ti
5. unemployment/
6. unemployment. ab, id, ti
7. employment status/
8. (employment status or employment or work resumption or working age or paid work or work functioning or job functioning). ab, id, ti
9. occupational status/
10. (occupational status or job status or work status or vocational status or work participation or employment participation or stay at work or presenteeism or work outcomes or work ability). ab, id, ti
11. employability/
12. (employability or work capacity or employment capacity). ab, id, ti
13. job satisfaction/
14. (job satisfaction or work retention or job retention). ab, id, ti
15. employee absenteeism/
16. (employee absenteeism or sick leave or absenteeism). ab, id, ti
17. 1 or 2 [chronic disorders]
18. or/3–16 [RTW or job retention]
19. 17 and 18

Note: no additional limits have been applied.

### CINAHL Plus with Full Text (EBSCOhost), Date of search 6 March 2014

- (MH “Chronic Disease”)
- SU chronic disease OR chronic disorder
- (MH “Job Re-Entry”)
- SU job re-entry
- MH “Unemployment”
- SU unemployment
- (MH “Employment+”)
- SU employment OR employment status OR working age
- (MH “Job Satisfaction+”)
- SU job satisfaction
- (MH “Sick Leave”)
- SU sick leave
- (MH “Absenteeism”)
- SU absenteeism
- (S1 OR S2)
- (S3 OR S4 OR S5 OR S6 OR S7 OR S8 OR S9 OR S10 OR S11 OR S12 OR S13 OR S14)
- S15 AND S16

Notes: no additional limits have been applied.

## Appendix 2

**Table:** Data-extraction of the six included studies

First author (year), country of origin	Study design	Participants: a) Sample size b) Gender (F %) c) Range of age (mean, SD) d) Chronic disease e) Employment at baseline (employed %)	Outcome: a) Definition b) Type of measurement	Factor: a) Definition b) Type of measurement	Effect (HR/OR/RR/correlation)	Quality assessment
Heijbel et al. (2006), Sweden	PC	a) 508 b) 90.9 c) 24–64 (50*, NR) d) Musculoskeletal pain (33.7 %**), mental distress (15.6 %**), respiratory disorders (1.9 %**), cardiovascular disorders (1.5 %**), other (i.e., neurological disorders, factures, diabetes, 12.0 %**), combination of disorders (35.2 %**) e) 100	a) RTW, defined as work status on the 18th month after baseline. Persons who were returned (part-time) and were working for <15 days during the 18th month were counted as returners b) Human resource departments in 5 municipalities and 4 county councils	a) Univariate logistic regression analyses: 1. Sex, male (r: female) 2. Age ≤44 years (r: 55–65 years) 3. Age 45–54 years (r: 55–65 years) 4. Own prediction of RTW, yes (r: no) 5. Complaints from >1 group of symptoms, no (r: yes) 6. Duration of the complaints ≤5 years (r: >5 years) 7. Duration of sick leave <1 year (r: ≥1 year) 8. Pain, 1st quartile (r: 4th quartile), measured by von Korff’s questionnaire 9. Pain, 2nd quartile (r: 4th quartile) 10. Pain, 3rd quartile (r: 4th quartile) 11. Function, 1st quartile (r: 4th quartile), measured by von Korff’s questionnaire 12. Function, 2nd quartile (r: 4th quartile) 13. Function, 3rd quartile (r: 4th quartile) 14. Physically strenuous work, no (r: yes) 15. Contact with the workplace/workmates, yes (r: no) 16. Perception of being welcome back to work, yes (r: no or do not know) 17. Contact with the Occupational Health Service, yes (r: no) 18. Contact with the Regional Social Insurance officer, yes (r: no) 19. Contact with the Trade Union, yes (r: no) 20. Rehabilitation programme, yes (r: no) b) Postal questionnaire (*n* = 520), telephone interview (*n* = 5)	OR [95 % CI]: 1. 1.23 [0.64, 2.39] **2. 2.48 [1.43, 4.31]** **3. 2.18 [1.30, 3.67]** **4. 15.99 [6.86, 37.25]** **5. 2.01 [1.29, 3.13]** **6. 1.75 [1.12, 2.72]** **7. 2.67 [1.76, 4.05]** **8. 3.73 [1.84, 7.55]** **9. 5.51 [2.74, 11.07]** **10. 2.23 [1.06, 4.70]** **11. 2.70 [1.41, 5.16]** **12. 2.05 [1.06, 3.97]** 13. 1.61 [0.82, 3.16] 14. 1.54 [1.00, 2.38] 15. 1.97 [0.94, 4.14] **16. 1.92 [1.23, 2.99]** 17. 1.35 [0.90, 2.02] 18. 0.84 [0.54, 1.31] 19. 1.08 [0.73, 1.61] 20. 1.12 [0.72, 1.75]	Sufficient
Baanders et al. (2002), the Netherlands	CS	a) 1,266 b) 56.5 c) 15–64 (NR, NR) d) Cardiovascular disease (7.7 %), chronic nonspecific lung diseases (18.6 %), locomotor disease (15.0 %), cancer (5.1 %), diabetes mellitus (10.4 %), neurological disease (9.1 %), digestive disorder (3.5 %), other (30.6 %) e) 45.1	a) WR, defined as labor market participation is defined as having a paid job for at least twelve hours per week b) Postal questionnaire	a) Not employed versus employed: 1. Gender, female (r: male) 2. Age, 25–44 years (r: 15–24 years) 3. Age, 45–64 years (r: 15–24 years) 4. Education level, primary (r: university) 5. Educational level, lower secondary + vocational (r: university) 6. Educational level, intermediate secondary + vocational (r: university) 7. Educational level, higher vocational (r: university) 8. Experiencing motor control problems, yes (r: no) b) Postal questionnaire.	B-coefficient***: **1.** ***p*** **<** **0.01, neg.** **2.** ***p*** **<** **0.01, pos.** 3. Nonsignificant **4.** ***p*** **<** **0.01, neg.** **5.** ***p*** **<** **0.01, neg.** 6. Nonsignificant 7. Nonsignificant **8.** ***p*** **<** **0.01, neg.**	Insufficient
Botticello et al. (2012), USA	CS	a) 1,013 b) 19.1 c) 17–64 (41.2, NR) d) Spinal cord injury; paraplegia (53.2 %), tetraplegia (46.8 %) e) 63.4	a) WR, defined as employment status, assessed at least one follow-up using the categories: working, homemaker, job-training program, sheltered workshop, student, retired, unemployed, or other (which includes volunteer work, disability, or medical leave). The variable was dichotomized as ‘paid employment’ ‘yes’ (category: ‘working’) and ‘no’ (remaining categories) b) Telephone interview	a) 1. >SES index (r: < SES index) defined as area-level socioeconomic index, combination of (1) employment rate, (2) percent of population residing within an urban area, (3) measures of household income, housing values, education and portion of residents employed in high status occupations 2. Rural (r: suburban), defined as <60 % of residents lived in an urban area (>50.000 residents) 3. Urban (r: suburban), defined as >90 % of residents lived in an urban area b) National Historical Geographical Information System, 2000 US Census Summary File	OR [95 % CI]: **1. 1.09 [1.04, 1.14]** 2. 0.53 [0.13, 2.20] **3. 0.46 [0.23, 0.93]**	Sufficient
Calsbeek et al. (2006), The Netherlands	CS	a) 246 b) 58.2** c) 15–24 (20.1**, NR) d) Inflammatory bowel disease (49.2 %), chronic liver diseases (11.7 %), congenital digestive disorder (17.6 %), food allergy (9.4 %), celiac disease (12.1 %) e) NR	a) WR, defined as labor participation, assessed by the number of hours employed per week which were dichotomized in ‘having a paid job’ (<12 h/w) vs. ‘not having a paid job) b) Postal questionnaire	a) Bivariate logistic regression analyses: 1. Physical complaints, yes (r: no) 2. Anxiety, yes (r: no) 3. Depression, yes (r: no) 4. Disability in endurance, yes (r: no) 5. Hospitalization, yes (r: no) 6. Use of medication, yes (r: no) 7. Need to diet adherence, yes (r: no) 8. Nocturnal toilet use, yes (r: no) b) Postal questionnaire	OR [95 % CI]: 1. 0.99 [0.94, 1.03] 2. 0.94 [0.88, 1.01] 3. 0.98 [0.89, 1.07] 4. 0.93 [0.80, 1.08] 5. 0.91 [0.80, 1.03] **6. 0.78 [0.62, 0.98]** 7. 1.04 [0.76, 1.43] **8. 0.70 [0.53, 0.91]**	Insufficient
Messmer Uccelli et al. (2009), 18 European countries	CS	a) 1,141 b) 67 c) 21–67 (41.8, 9.2) d) Multiple sclerosis e) 61	a) WR, defined as employment status; differentiating between employed or not employed b) Questionnaire	a) Category variables: 1. MS-related symptoms (more difficult, *n* = 20) 2. MS-related symptoms (easier, *n* = 7) 3. Workplace environment (more difficult, *n* = 13) 4. Workplace environment (easier, *n* = 19) 5. Your attitude toward work (more difficult, *n* = 6) 6. Your attitude toward work (easier, *n* = 4) 7. Attitudes of others in the workplace (more difficult, *n* = 6) 8. Attitudes of others in the workplace (easier, *n* = 4) 9. Financial considerations (more difficult, *n* = 5) 10. Financial considerations (easier, *n* = 4) 11. Personal considerations (more difficult, *n* = 9) 12. Personal considerations (easier, *n* = 9) Measured by the employed group, reference group is the unemployed group b) Questionnaire. Participants were asked to indicate to what extent each item made job maintenance easier or more difficult, from one of three options (1) not at all, (2) somewhat, (3) very much	OR [95 %]: **1. 0.96 [0.94, 0.98]** **2. 1.10 [1.04, 1.16]** 3. 0.97 [0.93, 1.10] **4. 1.04 [1.01, 1.06]** 5. 0.97 [0.90, 1.05] 6. 1.08 [0.99, 1.17] 7. 1.00 [0.92, 1.07] 8. 1.02 [0.95, 1.10] 9. 0.95 [0.89, 1.02] **10. 1.15 [1.07, 1.23]** 11. 1.00 [0.94, 1.06] 12. 0.98 [0.93, 1.03]	Insufficient
Muehrer et al. (2011), USA	RC	a) 102,104 b) NR c) 15–64 (NR, NR) d) Chronic kidney disease, end-stage renal disease (ESRD) e) NR	a) WR, defined as maintaining employment. Person’s inability to maintain employed was identified when, at initiation of treatment, persons changed employment from full- to part-time or to any other status (retired, student, homemaker, etc.) b) United States Renal Data System (USRDS)	a) Univariate logistic regression analyses (model 1999 through 2003): 1. Age, 15–19 years (r: 50–54 years) 2. Age, 20–24 years (r: 50–54 years) 3. Age, 25–29 years (r: 50–54 years) 4. Age, 30–34 years (r: 50–54 years) 5. Age, 35–39 (r: 50–54 years) 6. Age, 40–44 years (r: 50–54 years) 7. Age, 45–49 years (r: 50–54 years) 8. Age, 55–59 (r: 50–54 years) 9. Age, 60–64 (r: 50–54 years) 10. Gender, women (r: men) 11. Race, black (r: white) 12. Race, Asian (r: white) 13. Race, Native American (r: white) 14. Race, other (r: white) 15 Race, Hispanic (r: white) 16. ESDR cause, hypertension (r: diabetes) 17. ESRD cause, glomerulonephritis (r: diabetes) 18. ESRD cause, other (r: diabetes) 19. ESRD cause, cystic kidney (r: diabetes) 20. ESRD cause, other urologic (r: diabetes) 21. Inability to transfer, yes (r: no) 22. Alcohol use, yes (r: no) 23 Coronary vascular disease, yes (r: no) 24. Use of drugs, yes (r: no) 25. Cancer, yes (r: no) 26. Congestive heart failure, yes (r: no) 27. Tobacco use, yes (r: no) 28. Pericarditis, yes (r: no) 29. Arrhythmia, yes (r: no) 30. Inability to ambulate, yes (r: no) 31. Ischemic heart disease, yes (r: no) 32. COPD, yes (r: no) 33. Cardiac arrest, yes (r: no) 34. Diabetes (no insulin), yes (r: no) 35. Peripheral vascular disease, yes (r: no) 36. Anemia, yes (r: no) 37. Hypertension, yes (r: no) 38. Diabetes (insulin), yes (r: no) 39. Myocardial infarction, yes (r: no) 40. Predialysis erythropoietin use, yes (r: no) 41. Dialysis setting, home (r: dialysis facility) 42. Dialysis setting, hospital inpatient (r: dialysis facility) 43. Primary type of dialysis, continuous ambulatory peritoneal dialysis (r: hemodialysis) 44. Primary type of dialysis, continuous cycling peritoneal dialysis (r: hemodialysis) 45. Primary type of dialysis, intermittent peritoneal dialysis (r: hemodialysis) 46. Primary type of dialysis, other (r: hemodialysis) 47. Medicaid, yes (r: no) 48. Department of Veterans Affairs, yes (r: no) 49. Medicare, yes (r: no) 50. Insurance by employer, yes (r: no) 51. Other, yes (r: no) 52. No insurance (r: having an insurance) b) United States Renal Data System (USRDS)	OR [95 %]: 1. 0.79 [0.57, 1.12] **2. 0.85 [0.75, 0.97]** 3. 0.97 [0.88, 1.07] 4. 1.10 [0.92, 1.09] 5. 1.02 [0.95, 1.11] 6. 0.98 [0.91, 1.05] 7. 1.05 [0.98, 1.12] **8. 0.87 [0.82, 0.93]** **9. 0.89 [0.82, 0.97]** **10. 0.78 [0.74, 0.81]** **11. 0.75 [0.72, 0.78]** **12. 0.82 [0.74, 0.90]** 13. 0.99 [0.84, 1.17] **14. 0.85 [0.73, 0.98]** **15. 0.68 [0.65, 0.71]** 16. 1.02 [0.95, 1.10] **17. 1.22 [1.13, 1.32]** **18. 0.85 [0.78, 0.92]** **19. 1.68 [1.50, 1.87]** **20. 1.17 [1.00, 1.38]** 21. 0.85 [0.54, 1.34] **22. 0.56 [0.47, 0.67]** **23. 0.70 [0.62, 0.79]** **24. 0.73 [0.60, 0.89]** **25. 0.73 [0.65, 0.81]** **26. 0.80 [0.76, 0.85]** **27. 0.85 [0.79, 0.93]** 28. 0.97 [0.77, 1.21] 29. 0.88 [0.76, 1.02] **30. 0.45 [0.35, 0.58]** **31. 0.92 [0.85, 0.99]** **32. 0.86 [0.75, 0.98]** **33. 0.55 [0.39, 0.76]** 34. 1.02 [0.96, 1.08] 35. 0.94 [0.86, 1.02] 36. 1.01 [0.99, 1.02] **37. 1.06 [1.00, 1.11]** **38. 0.94 [0.89, 1.00]** 39. 0.94 [0.84, 1.05] **40. 1.29 [1.23, 1.35]** 41. 1.01 [0.89, 1.15] **42. 1.13 [1.01, 1.26]** **43. 1.40 [1.23, 1.59]** **44. 1.61 [1.40, 1.87]** 45. 1.47 [0.64, 3.37] 46. 0.90 [0.13, 6.10] **47. 0.39 [0.35, 0.43]** **48. 0.80 [0.65, 0.99]** **49. 0.81 [0.74, 0.88]** **50. 1.51 [1.36, 1.67]** **51. 1.36 [1.23, 1.50]** **52. 0.43 [0.38, 0.48]**	Insufficient

The multivariate model is reported, unless stated otherwise

Bold values indicate all significant factors measured from p < 0.05

*NR* not reported, *r* reference category, *PC* prospective cohort, *RC* retrospective cohort, *CC* case–control study, *CS* cross-sectional study

* Median of population at baseline

** Statistics of population at baseline

*** Relative change in probability of working. No other effect measurement (HR/OR/RR/correlation) reported

## Footnotes

1
^a^OR not available for this study
